# Dual Functions of ASCIZ in the DNA Base Damage Response and Pulmonary Organogenesis

**DOI:** 10.1371/journal.pgen.1001170

**Published:** 2010-10-21

**Authors:** Sabine Jurado, Ian Smyth, Bryce van Denderen, Nora Tenis, Andrew Hammet, Kimberly Hewitt, Jane-Lee Ng, Carolyn J. McNees, Sergei V. Kozlov, Hayato Oka, Masahiko Kobayashi, Lindus A. Conlan, Timothy J. Cole, Ken-ichi Yamamoto, Yoshihito Taniguchi, Shunichi Takeda, Martin F. Lavin, Jörg Heierhorst

**Affiliations:** 1 St. Vincent's Institute of Medical Research, Fitzroy, Australia; 2 Department of Medicine, St. Vincent's Hospital, The University of Melbourne, Fitzroy, Australia; 3 Department of Biochemistry and Molecular Biology and Department of Anatomy and Developmental Biology, Monash University, Clayton, Australia; 4 Queensland Institute of Medical Research, Herston, Australia; 5 Department of Radiation Genetics, Graduate School of Medicine, Kyoto University, Kyoto, Japan; 6 Cancer Research Institute, Kanazawa University, Ishikawa, Japan; 7 Central Clinical Division, University of Queensland, Royal Brisbane Hospital, Herston, Australia; Duke University Medical Center, United States of America

## Abstract

Zn^2+^-finger proteins comprise one of the largest protein superfamilies with diverse biological functions. The ATM substrate Chk2-interacting Zn^2+^-finger protein (ASCIZ; also known as ATMIN and ZNF822) was originally linked to functions in the DNA base damage response and has also been proposed to be an essential cofactor of the ATM kinase. Here we show that absence of ASCIZ leads to *p53*-independent late-embryonic lethality in mice. *Asciz*-deficient primary fibroblasts exhibit increased sensitivity to DNA base damaging agents MMS and H_2_O_2_, but *Asciz* deletion or knock-down does not affect ATM levels and activation in mouse, chicken, or human cells. Unexpectedly, *Asciz*-deficient embryos also exhibit severe respiratory tract defects with complete pulmonary agenesis and severe tracheal atresia. Nkx2.1-expressing respiratory precursors are still specified in the absence of ASCIZ, but fail to segregate properly within the ventral foregut, and as a consequence lung buds never form and separation of the trachea from the oesophagus stalls early. Comparison of phenotypes suggests that ASCIZ functions between Wnt2-2b/ß-catenin and FGF10/FGF-receptor 2b signaling pathways in the mesodermal/endodermal crosstalk regulating early respiratory development. We also find that ASCIZ can activate expression of reporter genes via its SQ/TQ-cluster domain *in vitro*, suggesting that it may exert its developmental functions as a transcription factor. Altogether, the data indicate that, in addition to its role in the DNA base damage response, ASCIZ has separate developmental functions as an essential regulator of respiratory organogenesis.

## Author Summary

ASCIZ is a DNA damage response protein that has been proposed to be a regulator and stabilizing co-factor of the ATM kinase, mutations of which lead to a syndrome involving neurological and immune dysfunctions, tumour predisposition, and X-ray hypersensitivity. To study *Asciz* function *in vivo*, we have generated a knockout mouse model lacking this gene. Here we show that ASCIZ has a specific role in mediating cell survival in response to DNA base damage, but it is not required for stabilization and regulation of ATM. Strikingly, *Asciz* knockout mice fail to survive to birth and have tissue-specific defects in embryonic development. In particular, *Asciz* null embryos fail to develop lungs and undergo an early arrest in tracheal development. The precursor cells that normally form the lung are present in our embryos, but they fail to segregate from the foregut. These observations indicate that ASCIZ plays an important and previously unrecognized developmental role that is most likely unrelated to its function in mediating responses to DNA damage. Our study delineates the function of ASCIZ in DNA damage survival and highlights an exciting new function of the protein in controlling the early stages of lung development.

## Introduction

Pathways that maintain genome integrity by responding to spontaneous DNA damage are crucial for normal development and ageing, and act as tumor suppressors to prevent the onset of cancer [1]. While DNA damage signaling is rather generic in that structurally diverse lesions eventually lead to activation of one or both of the central checkpoint kinases ATM or ATR [2], DNA repair pathways are believed to be highly lesion-specific [1]. In addition to environmental DNA damage, eukaryotic cells incur a high level of spontaneous DNA damage as a consequence of normal metabolism, most notably abasic sites that are generated as repair intermediates of the base excision repair (BER) pathway with an estimated incidence of ∼10,000 per cell per day [3]. Abasic sites can emanate from various base modifications (e.g. oxidation, methylation), which for experimental purposes are most commonly generated by treatment with methylmethane sulfonate (MMS) or H_2_O_2_, and key BER enzymes for their repair include apyrimidinic/apurinic endonuclease (APE) and DNA polymerase beta (Polß) [4], [5].

The importance of the BER pathway is indicated by findings that absence of any of the BER genes acting downstream of abasic sites results in embryonic or perinatal lethality in mice [6], [7]. However, some of the key BER enzymes also seem to have DNA damage-independent functions; for example, APE1 has a separate role as a redox regulator of several transcription factors [8]. Similarly, increased apoptotic cell death during development of *Polß*-null mice can be suppressed by deletion of *p53 (TRP53)*, indicating that this part of the phenotype is indeed due to defective base damage repair. On the other hand, the perinatal lethality of these mice that is associated with defective neuronal and lung development as a DNA damage-independent defect is not rescued by *p53* deletion [9]–[11].

While the DNA damage processing enzymes of the BER pathway are clearly defined, new accessory factors that regulate the activity or stability of Polß and other BER enzymes keep emerging, including the non-histone DNA-binding protein HMGB1 [12], arginine methyl-transferases [13], and ubiquitin ligases [14]. We recently identified ASCIZ ( = ATM substrate Chk2-interacting Zn^2+^-finger) as a new Zn^2+^-finger (ZnF) protein with roles in the DNA base damage response. In human cells, ASCIZ forms DNA damage-induced nuclear foci specifically in response to DNA damaging agents that generate lesions repaired by the BER pathway (MMS and H_2_O_2_) in a manner that is enhanced by the BER inhibitor methoxyamine, and *Asciz* depletion by siRNA leads to increased MMS sensitivity [15]. Likewise, *Asciz* deletion in the chicken DT40 B lymphocyte line leads to increased sensitivity to MMS and H_2_O_2_, but not to ionizing radiation (IR), UV irradiation and other DNA lesions, as well as increased erroneous repair of enzyme-generated DNA base damage consistent with a role in the BER pathway [16]. Moreover, *Asciz* deletion suppresses the dramatic MMS hypersensitivity of Polß-deficient DT40 cells [16], reminiscent of the protective effect of simultaneous deletion of the relevant upstream methyl-purine-glycosylase (MPG) in Polß-deficient murine embryonic fibroblasts [17]. ASCIZ contains a large number of conserved ATM/ATR kinase phosphorylation sites in an SQ/TQ cluster domain [18], and consistent with its original classification as an ATM substrate, ASCIZ was subsequently re-isolated as an ATM-interacting protein (thus also called ATMIN)[19]. It was proposed that ASCIZ acts as an essential co-factor of ATM that was required for ATM stability (and vice versa ATM was required for ASCIZ stability) as well as for ATM activation by some stimuli, though surprisingly not by canonical DNA damaging ATM activators such as IR [19].

To better understand the role of ASCIZ *in vivo*, we have here generated a mouse line that lacks the vast majority of the *Asciz* protein-coding sequence in the germline. Our results confirm that *Asciz*-deficient cells are specifically hypersensitive to DNA lesions that are processed by the BER pathway, but challenge the proposed interdependence between ASCIZ and ATM levels. In contrast to *Atm*-deficient mice that overall develop normally [20], *Asciz* deletion results in late embryonic lethality with severe respiratory defects reminiscent of mouse mutants in Wnt2/2b and FGF10 signaling pathways. The data indicate that *Asciz* has an unexpected DNA damage-independent developmental function as an essential regulator of pulmonary organogenesis.

## Results

### Generation of *Asciz* gene-targeted mice

**Figure 1:**
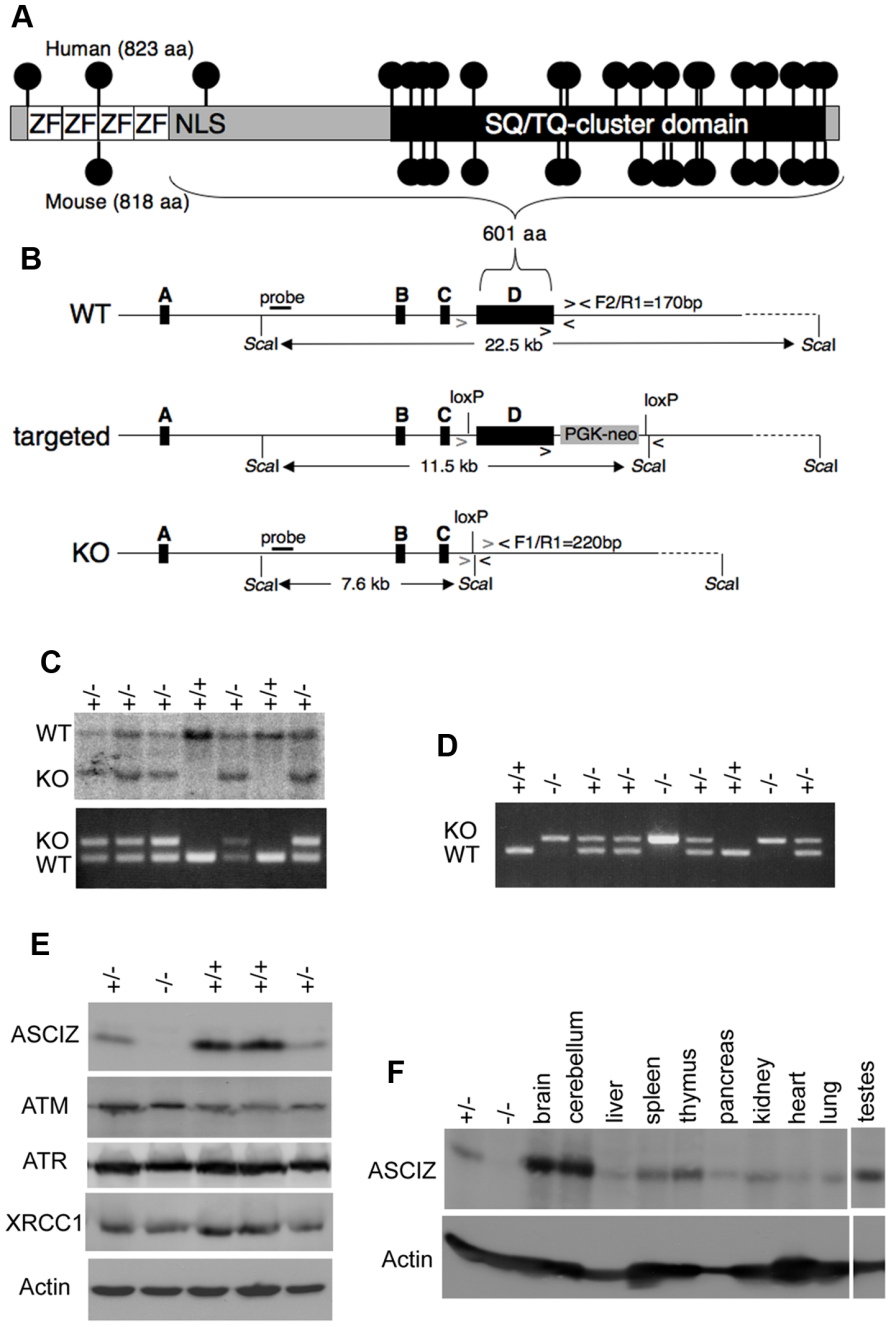
Generation of *Asciz*-deficient mice. (A) Schematic comparison of human and mouse ASCIZ. ZF, Zn^2+^ finger; NLS, nuclear localization signal. Lollipops indicate predicted ATM/ATR phosphorylation sites. (B) *Asciz* gene structure and targeting strategy, drawn approximately to scale. The four exons (A–D) are indicated by black boxes, as are locations of oligonucleotide primers, *Sca*I restriction sites and the probe for genotyping, and the positions of loxP sites. (C) Southern blot (top) and PCR genotyping (bottom) of a randomly chosen litter from a heterozygote intercross at weaning. (D) PCR genotyping of a randomly chosen litter at E15.5. (E) Western blot analysis of head extracts of a randomly chosen litter at E12.5 using the indicated antibodies. (F) Western blot analysis of the indicated tissues of an 8-week old male WT mouse, and E15.5 *Asciz^+/−^* and *Asciz^−/−^* head extracts as antibody specificity controls.

Human and mouse *Asciz* have a similar gene structure where exons A–C encode the N-terminal ZnF region of about 220 amino acid residues, and exon D encodes the bulk of the protein (601 of 823 or 818 residues) including the nuclear localization signal, core domain and SQ/TQ cluster domain (Figure 1A, 1B; NCBI Gene ID 23300). Because there is evidence for expression of alternative isoforms that differ in the number of N-terminal ZnFs (http://www.uniprot.org/uniprot/O43313), we integrated loxP sites flanking exon D into the murine *Asciz* locus to remove the majority of the protein-coding sequence (Figure 1B). Germline deletion of this exon after crossing with *PGK-Cre* knock-in mice, followed by outcrossing of *PGK-Cre* (all on a pure C57BL/6 background), was confirmed by Southern blot and PCR genotyping (Figure 1C). In over 600 offspring from *Asciz^+/−^* heterozygote intercrosses genotyped at weaning (∼3 weeks of age), we failed to detect any homozygous *Asciz*-deleted mice (Figure 1C and Table 1). However, homozygous *Asciz*-deleted embryos were readily detectable even at relatively late stages of gestation (Figure 1D; and more detail below).

**Table 1:** Genotypes of *Asciz^+/−^* intercross litters.

age	*Asciz^+/+^*	*Asciz^+/−^*	*Asciz^−/−^* (%)	*Asciz^−/−^* alive	*Asciz^−/−^* exencephaly
weaning	270	418	0	0	-
E18.5	9	9	4 (18)	0	1
E16.5	25	36	13 (18)	8	5
E15.5	13	15	8 (24)	6	2
E14.5	29	60	22 (20)	18	7
E13.5	10	25	9 (20)	8	3
E12.5	32	53	13 (13)	12	2
E11.5	23	32	15 (21)	15	2
E8.5–10.5	29	44	30 (29)	n.d.	n.d.

n.d., not determined.

Western blotting of head extracts confirmed the absence of ASCIZ protein in *Asciz^−/−^* embryos, and a ∼50% reduction of protein levels in heterozygotes compared to wildtype (WT) littermates (Figure 1E). Levels of other DNA damage response proteins (including ATM) appeared to be normal in *Asciz*-deficient embryos (Figure 1E and below). In Northern blots using a probe for the non-deleted exon C, the residual exon D-deleted *Asciz* transcript was present in homozygous targeted embryos at <15% of wildtype (WT) mRNA levels (Figure S1), indicating that the mutated mRNA is highly unstable. Using *Asciz* null embryo lysates as an antibody specificity control, we found that ASCIZ is ubiquitously expressed in adult mice, with overall similar levels relative to the loading control in all tissues except for somewhat higher levels in the brain, cerebellum and testes (Figure 1F).

### Absence of *Asciz* leads to *p53*-independent late-embryonic lethality

**Figure 2:**
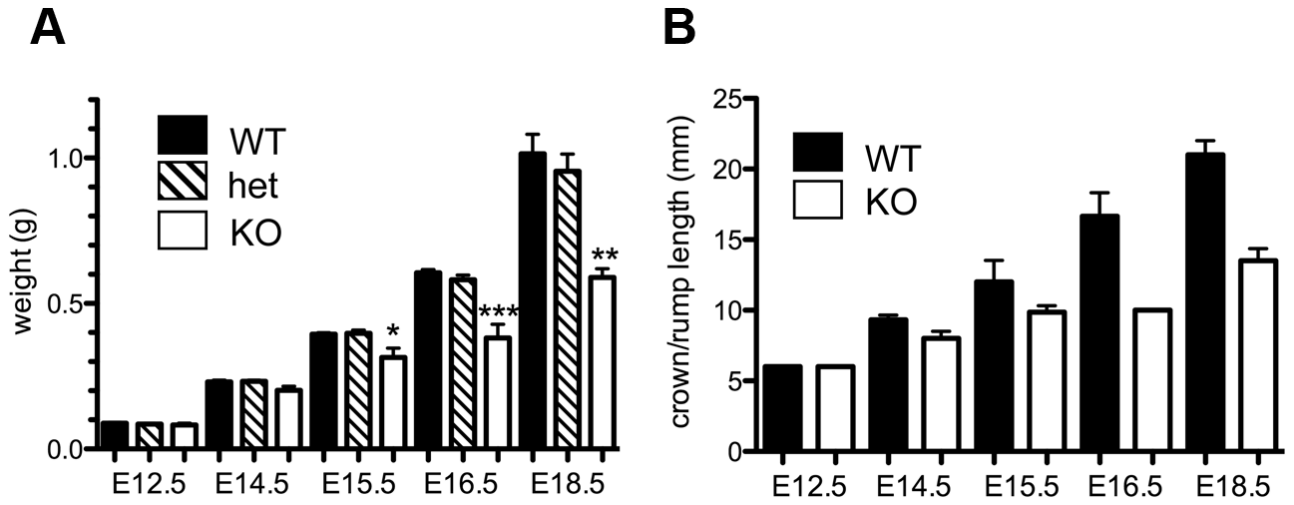
Late gestational growth defect of *Asciz*-deficient embryos. (A) Embryo weights of WT, heterozygotes and *Asciz*-deficient embryos at the indicated times post-conception. Data are the mean ± standard error; 22–55 embryos were analysed per timepoint (KOs: n = 8 (E12.5), 12 (E14.5), 8 (E15.5), 11 (E16.5), and 4 (E18.5). (B) Crown-rump length of embryos determined by histomorphometry. Data are mean ± standard error, n = 3–9 per data point. *p<0.05, **p<0.01, ***p<0.001.

The absence of homozygous *Asciz^−/−^* mice at weaning prompted us to investigate the development of ASCIZ-deficient embryos in more detail. *Asciz^−/−^* embryos were recovered at near-Mendelian ratios at all time points analysed (Table 1). Based on peripheral circulation scored during uterine dissections, *Asciz^−/−^* embryos appeared to lose viability around embryonic day 16.5 post conception (E16.5) (Table 1), at which point they were considerably growth-retarded compared to littermates (Figure 2A, 2B). Embryonic lethality due to DNA damage response gene deletions can often be suppressed by *p53* deletion [6]. To test if *p53* status affects the essential requirement for *Asciz*, we intercrossed compound *Asciz^+/−^/p53^+/−^* heterozygous mice. However, we could again not detect any viable *Asciz^−/−^* mice amongst >300 genotyped offspring at weaning (Table 2). Altogether, these data indicate that absence of *Asciz* leads to progressively impaired development during late gestation and becomes absolutely incompatible with life a few days before term.

**Table 2:** Genotypes of offspring from *Asciz^+/−^ p53^+/−^* intercrosses at weaning.

	*Asciz^+/+^*	*Asciz^+/−^*	*Asciz^−/−^*
***p53^+/+^***	36	69	0
***p53^+/−^***	49	107	0
***p53^−/−^***	16	25	0

### *Asciz* deficiency leads to modestly accelerated senescence and BER-like DNA damage hypersensitivity in primary fibroblasts

**Figure 3:**
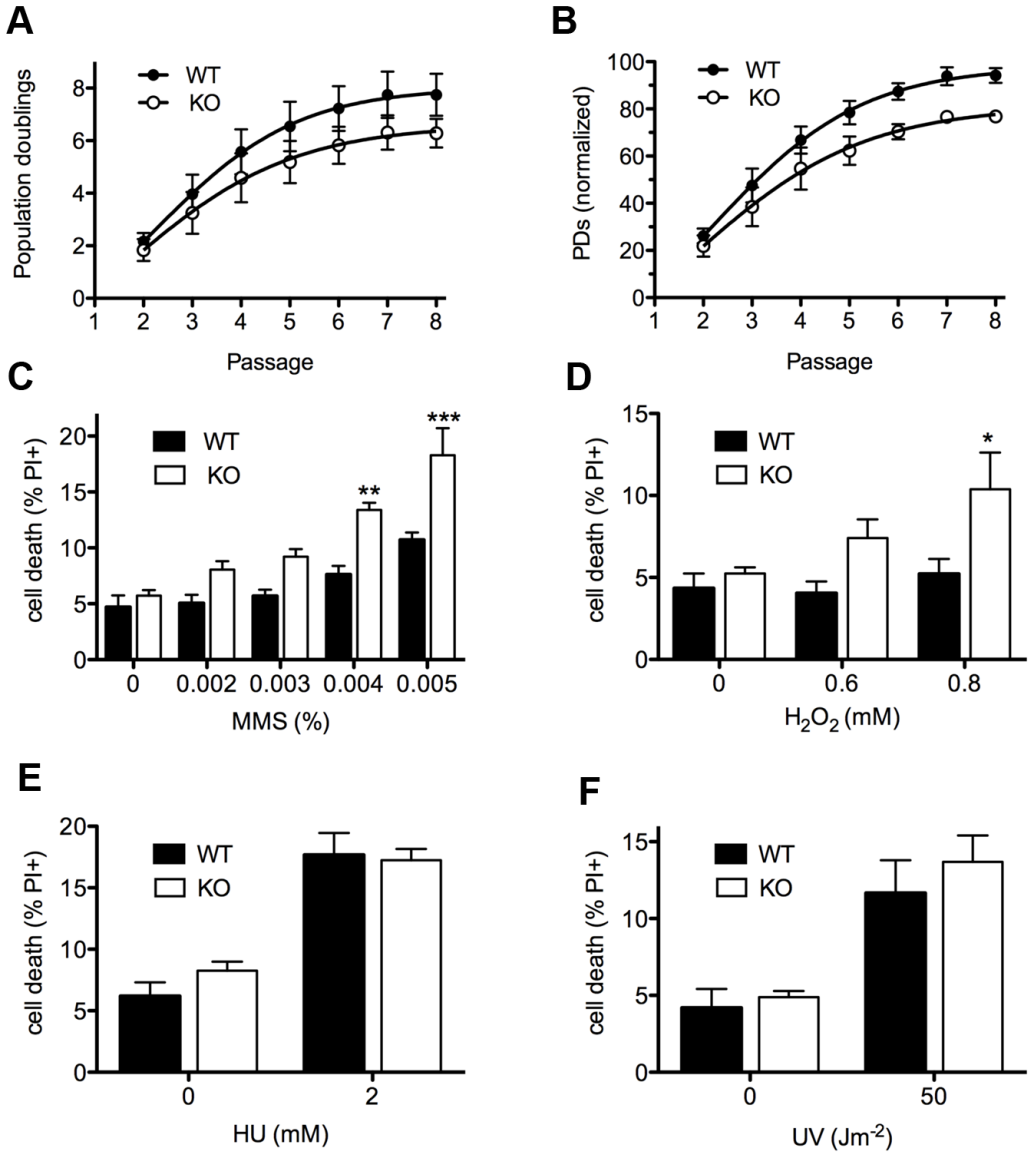
Cellular phenotypes of *Asciz*-deficient primary fibroblasts. (A) Cumulative population doublings (PDs) of *Asciz*-deficient and matched WT littermate MEFs in a standardized 3T3 assay. Data are mean ± standard error, n = 6. (B) Data from panel A normalized to the PD maximum in the relevant litter. (C–F) DNA damage sensitivity assays. Cells were treated with the indicated doses of MMS, H_2_O_2_, 2 mM HU or 50 J/m^2^ UV, and viability was determined by propidium iodide exclusion and FACS. *p<0.05, **p<0.01, ***p<0.001.

To monitor DNA damage sensitivity of primary *Asciz*-deficient cells, we isolated murine embryonic fibroblasts (MEFs) from viable *Asciz^−/−^* embryos and matched WT littermate controls between E12.5–E14.5 (i.e., before growth retardation was apparent). Standardized proliferation assays using a 3T3 protocol under normoxic conditions (20% O_2_) revealed a modest premature senescence phenotype of *Asciz*-deficient MEFs compared to WT controls, with growth arrest after ∼20% fewer population doublings (Figure 3A). When normalized to the maximum population doublings within each litter, *Asciz^−/−^* MEFs always senesced earlier than the matched WT cultures (Figure 3B). As senescence of MEFs under these conditions is believed to involve an oxygen-induced DNA damage response [21], these results indicated a role of ASCIZ in the response to oxidative base damage in primary cells. To corroborate this, we treated early-passage MEFs (P2–P3, when proliferation differences between genotypes were minimal) with a panel of DNA damaging agents. In these assays, *Asciz*-deficient MEFs were significantly more sensitive to MMS and H_2_O_2_, which cause damage that is predominantly repaired by the BER pathway, compared to matched WT littermate controls (Figure 3C, 3D), but they were not hypersensitive to agents such as UV or hydroxyurea (HU) whose damage is repaired by other pathways (Figure 3E, 3F). MMS hypersensitivity of *Asciz* ^−/−^ MEFs was less pronounced than that of WT cells co-treated with methoxyamine (Figure S2), which blocks the single-nucleotide BER pathway through partial inhibition of APE1 and Polß [5]. In addition, MMS hypersensitivity of ASCIZ-deficient cells was further enhanced by methoxyamine (Figure S2), indicating that absence of ASCIZ only partially impairs BER. Altogether, these results are consistent with a role of *Asciz* as an accessory factor in the BER pathway in primary cells.

Because we had originally identified ASCIZ based on its interaction with Chk2 [15], and because ASCIZ was later proposed to be sometimes required for ATM activation [19], we tested if the MMS hypersensitivity of *Asciz^−/−^* MEFs could be due to defective ATM signaling. However, there was no reduction in MMS-dependent ATM activation (detected by an antibody against mouse pS1987-ATM; human pS1981-ATM) and phosphorylation of key ATM/ATR targets γH2AX and pS18-p53 in *Asciz*-deficient MEFs compared to WT littermate controls (Figure S3A, S3B). MMS-induced p53-S18 phosphorylation was completely abolished by the highly specific synthetic ATM kinase inhibitor KU55933 [22] (Figure S3C), indicating that it is a genuinely ATM-dependent process, whereas H2AX phosphorylation under these conditions was ATM-independent and thus likely ATR-mediated. Because our antibody for Chk2 phosphorylation on T68, widely considered to be one of the most ATM-specific phosphorylation sites, did not detect this site in MEF extracts (data not shown), we monitored Chk2-T68 phosphorylation in human U2OS cells following *Asciz* depletion by siRNA treatment. However, Chk2 was still efficiently phosphorylated in response to MMS (as well as IR) in *Asciz*-depleted cells (Figure S4A, S4B). Altogether, these data indicate that the increased MMS sensitivity of *Asciz*-deficient cells is not caused by impaired ATM signaling.

### ASCIZ and ATM protein levels are not interdependent in mouse, human, or chicken cells

**Figure 4:**
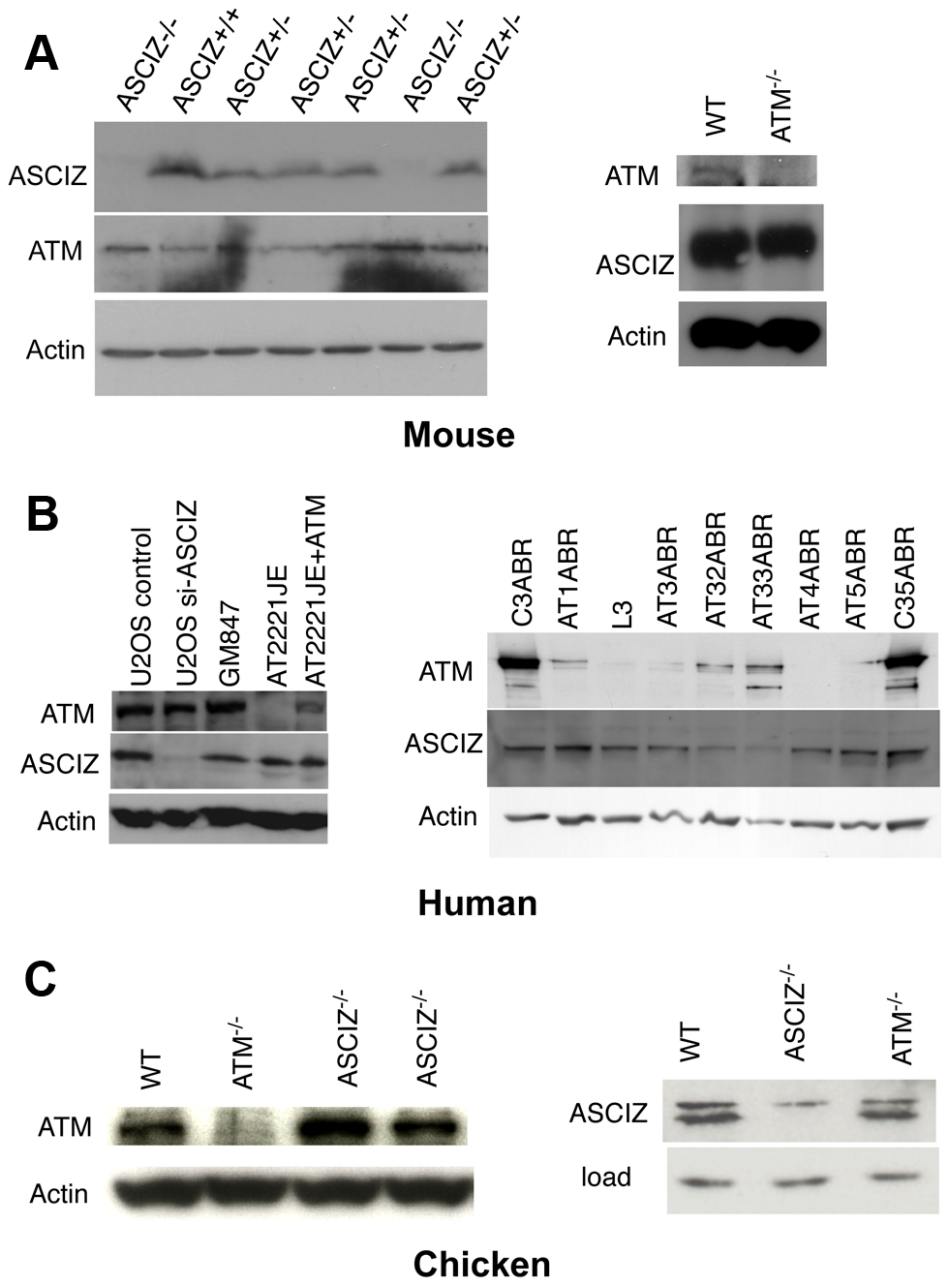
Reciprocal independence of ASCIZ and ATM protein levels. (A) Protein levels in mouse tissues. Left panel, Western blot analysis of head extracts of a randomly chosen litter from an *Asciz* heterozygote intercross at E12.5. Right panel, brain extracts of WT and *Atm*-null littermate mice [20]. (B) Protein levels in human cell lines. Left panel, adherent cells: U2OS osteosarcoma cells treated with *GL2* control or *Asciz* siRNA; GM847 control fibroblasts, *Atm*-deficient AT2221JE fibroblasts containing an empty-vector control (FTY pEBS7) or reconstituted with WT *Atm* (FTYZ5) [23]. Right panel, lymphoblastoid cell lines from healthy donors (C3ABR, C35ABR) and seven separate AT patients (L3 and AT1ABR–AT33ABR); note that ATM was immunoprecipitated before blotting as described [24]. (C) Protein levels in chicken DT40 B cell lysates. Left panel, comparison of ATM levels in two independent *Asciz*-deleted clones using the anti-chicken ATM antibody and the ATM-deleted DT40 clone as specificity control. Right panel, comparison of ASCIZ levels in WT and an *Atm*-deleted clone [25] with an *Asciz*-deficient clone [16] as antibody specificity control (NB, anti-human ASCIZ was used at 1∶100 dilution rather than 1∶2000–1∶4000 for mouse or human samples).

During analyses of DNA damage signaling in *Asciz*-depleted human cells (Figure S4B and data not shown) and initial protein blots of embryo extracts (Figure 1E), we could not detect any meaningful reduction of ATM levels in *Asciz*-deficient cells. Because this contradicted the recent report that ASCIZ and ATM levels were mutually dependent on each other [19], we explored this discrepancy first in additional embryos. Again, we saw no reduction of ATM protein levels in any of the *Asciz^−/−^* or heterozygous samples compared to WT littermate controls (Figure 4A, left panel, and data not shown). Likewise, we also did not see a reduction of ATM levels in human cells after almost complete depletion of ASCIZ by siRNA-treatment (Figure 4B, left panel, compare control siRNA treated to si-*ASCIZ* treated U2OS cells, lanes 1 and 2; and data not shown), or in two independently generated *ASCIZ* knockout clones [16] in the chicken DT40 B cell line (Figure 4C, left panel).

We also revisited the proposal that ATM was in turn required for ASCIZ stability [19]. In contrast to the severe reduction of ASCIZ levels in an ataxia telangiectsia (AT) fibroblast line reported by Kanu and Behrens (2007), we did not detect any loss of ASCIZ in another human AT patient-derived fibroblast cell line (Figure 4B, left panel, AT2221JE) that is considered to be *bona fide* ATM-deficient [23] compared to control fibroblasts (Figure 4B, left panel, GM847) or the isogenic AT cell line reconstituted with WT *Atm* (Figure 4B, left panel, AT2221JE+ATM [23]). We expanded this analysis to a panel of seven independent human AT-patient derived lymphoblastoid cell lines [24]. When adjusted for loading (actin), there was no reduction of ASCIZ levels in four of these lines that contained no or extremely low levels of residual ATM protein (AT1ABR, L3, AT4ABR, AT5ABR) compared to two independent healthy donor control cell lines (C3ABR, C35ABR)(Figure 4B, right panel); ASCIZ levels were also unaffected in two further AT lines that contained intermediate ATM protein levels (AT1ABR, AT33ABR), and there was only a modest reduction of ASCIZ levels in a third AT cell line with intermediate ATM levels (AT32ABR). Similarly, there was also no reduction of ASCIZ protein levels in brain lysates of *Atm* null mice [20] compared to WT littermates (Figure 4A, right panel), or in an *Atm*-deleted chicken DT40 clone [25] compared to the WT control (Figure 4C, right panel). Taken together, these data demonstrate that ASCIZ and ATM are not required for each other's stability in three different vertebrate species.

### *Asciz* is essential for pulmonary organogenesis

**Figure 5:**
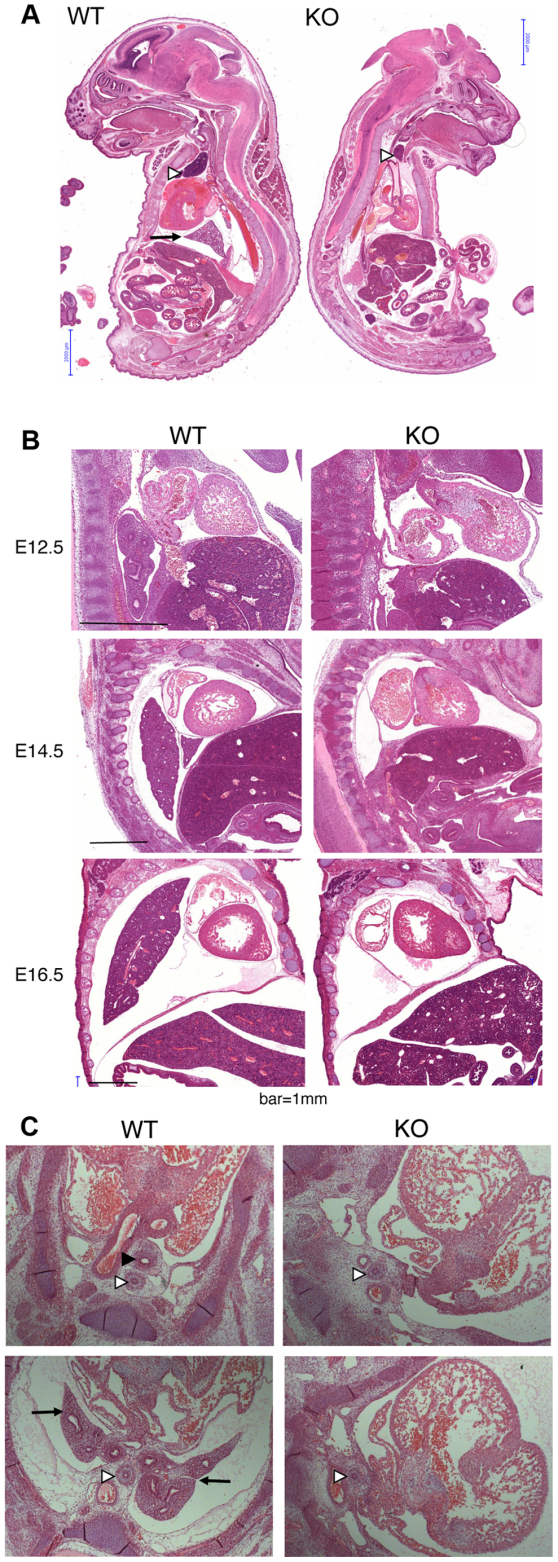
Histological analysis of *Asciz*-null embryos. (A) Sagittal sections of comparable levels of WT and *Asciz^−/−^* littermates at E18.5. Note the absence of lung (arrow), hypoplastic thymus (arrowhead), compressed thorax, steep ascending aorta, and exencephaly in the *Asciz*-null embryo. This embryo also represents an isolated case of omphalocele. Scale bars = 2 mm. (B) Micrographs of comparable sagittal sections of WT and *Asciz^−/−^* littermates at E12.5–16.5. Note the apparent caudal drop of the atrium relative to the ventricle in *Asciz* null embryos compared to WT littermates where the atrium seems to be propped up by the developing left lung. Scale bars = 1 mm. (C) Micrographs of comparable transverse sections of E12.5 WT and *Asciz^−/−^* littermates at the upper (top panels) and lower levels (bottom panels) of the thorax. Open arrowheads point to the oesophagus, the filled arrowhead and arrows point at the trachea and lungs respectively that are only present in the WT.

Because of the overall relatively mild DNA damage phenotypes of *Asciz*-deficient primary cells (Figure 3) and the absence of a *p53*-effect on viability (Table 2), we wondered whether the underlying cause for the late gestational lethality of *Asciz^−/−^* embryos could be DNA damage-independent, and performed histological analyses of litters between E12.5 and E18.5. The most striking defect at all time points was the complete absence of lungs in all *Asciz*-deficient embryos analyzed (n>30; Figure 5A–5C) and apparent lack of tracheal tissue in all but one of these (Figure 5C and data not shown); consistently, in all cases where absence of lungs was subsequently noticed during routine MEF or protein preparations, this phenotype was 100% predictive of the *Asciz^−/−^* genotype (not shown).

Interestingly, the absence of lungs seemed to lead to topological alterations in the position of the heart and its axis within the thoracic cavity, with an apparent drop of the atrium in *Asciz* null embryos into the space otherwise occupied by the lung in WT littermates (Figure 5B, 5C). In addition, the thymus appeared hypoplastic in all *Asciz^−/−^* embryos analyzed (Figure 5A), which could also be a secondary consequence of the defective respiratory system as the thymus descends into the mediastinum from its common origin with the parathyroid gland in close proximity of the upper trachea [26]. Besides these thoracic defects, ∼25% of *Asciz^−/−^* embryos exhibited already macroscopically obvious exencephaly (Figure 5A and Table 1), indicating that ASCIZ also contributes to neural tube development, but histologically other organs seemed to be developing normally.

### ASCIZ is required for separation of respiratory progenitors from the ventral foregut

The combined trachea and lung defects in *Asciz^−/−^* embryos were interesting because both organs originate at the same time but presumably independently of each other from the common respiratory endoderm in the ventral foregut [27], [28]. Shortly after specification of respiratory precursors that are characterized by expression of the Nkx2.1 transcription factor, bilateral lung buds and, just rostral of these, a central tracheal primordium emerge from the ventral foregut around E9.5 in the mouse. The junction of these primordia marks the bifurcation of the trachea into the two main bronchi, and the lung buds expand caudally into the surrounding mesoderm to form the bronchial tree and pulmonary epithelium by branching morphogenesis, whereas the trachea septates from the common foregut lumen in an upwards “unzipping” motion [27], [28]. Thus, in a simplified view, the origin of the respiratory system can be traced back to the projection of the tracheo-bronchial bifurcation onto the ventral oesophagus.

**Figure 6:**
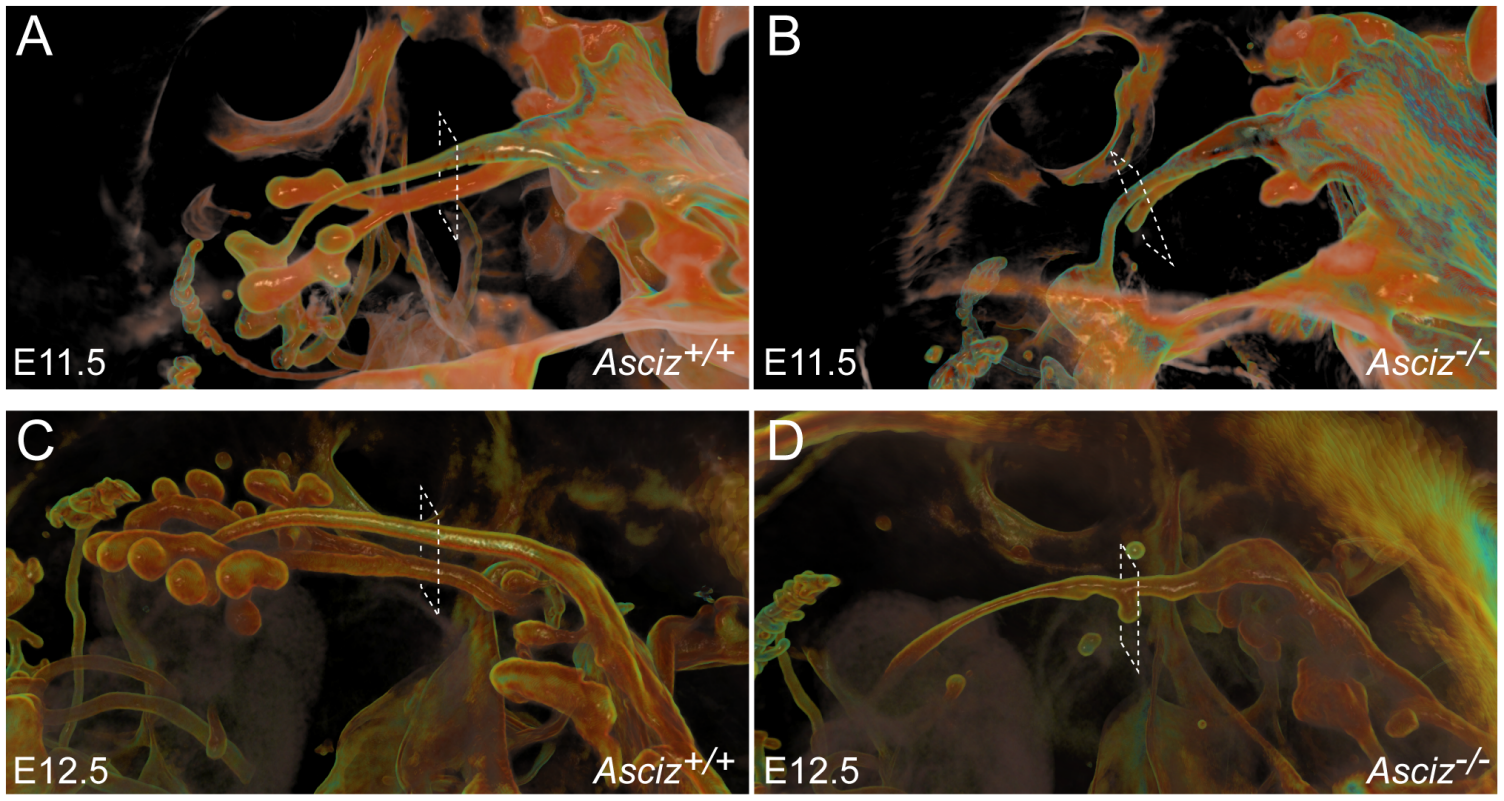
Defective pulmonary and tracheal development in *Asciz*-null embryos. Optical projection tomography of whole-mount E-cadherin stained of E11.5 (A, B) and E12.5 (C, D) littermates. Stippled boxes indicate the approximate plane of sections chosen for immunofluorescence analysis in Figure 7. Panels are arranged with the oesophagus on top.

To more clearly assess the developing trachea and lungs in three dimensions, we performed optical projection tomography (OPT) on whole-mount E-cadherin stained embryos. WT embryos showed clear separation of oesophagus and trachea at the larynx, bifurcation of the trachea into two bronchi and advanced branching of the developing pulmonary epithelium (Figure 6A, 6C). As expected, all five *Asciz^−/−^* embryos analyzed again lacked developing pulmonary epithelium (Figure 6B, 6D, Figure S5, and data not shown). One *Asciz* null embryo contained a very short incompletely separated tracheal stump that ended bluntly where it would normally connect to the main bronchi (Figure 6B). Interestingly, the other *Asciz* null embryos contained single centrally located bud-like structures that emerged from the ventral oesophagus near the level where the trachea bifurcates into bronchi in the relevant WT littermates (Figure 6D, Figure S5); the central location suggested that this bud-like structure represented tracheal primordium.

**Figure 7:**
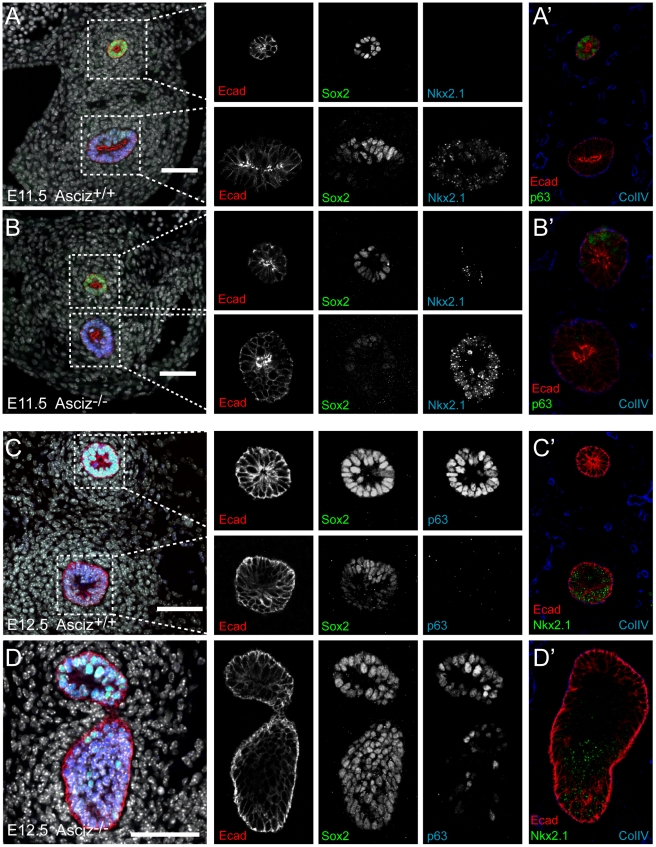
Expression analysis of markers of foregut development. Sections from the levels indicated in Figure 6 were stained with the indicated antibodies. All panels are oriented with the oesophagus or dorsal foregut on top. A′–D′ are sections adjacent to the ones shown in A–D. In the merged panel on the left, nuclei are counterstained with DAPI. Scale bars = 20 µm.

Two of the *Asciz^−/−^* whole-mount embryos and littermate controls were sectioned at the level of the truncated trachea (Figure 7B, 7B′) or tracheal bud-like structure (Figure 7D, 7D′) for immunofluorescence staining with the respiratory marker Nkx2.1. The tracheal stump in the mutant stained homogenously with Nkx2.1 (Figure 7B, bottom panel), similar to the trachea in the WT littermate (Figure 7A), and the ventral part of the tracheal bud-like structure in the other *Asciz^−/−^* embryo was also enriched for Nkx2.1 (Figure 7D′) with staining intensity similar to the separated trachea in the matched WT littermate control (Figure 7C′). Interestingly, in stark contrast to the WT oesophagus, some ectopic Nkx2.1-positive cells remained in the ventral part of the oesophagus in the mutant where the trachea had partially separated (Figure 7B, top panel).

We also analysed these sections for expression of p63, a p53-like transcription factor that is normally highly expressed in the oesophagus, but also present in basal cells of the trachea [29]. Under our staining conditions at the developmental stages studied here, p63 seemed only to be present in the oesophagus but not in the trachea in WT embryos (Figure 7A′, 7C). However, p63-positive cells were readily detectable in the ventral part of the tracheal bud-like structure in the *Asciz* ^−/−^ embryo (Figure 7D), suggesting defective partitioning of specified cells between trachea and oesophagus. As ectopic p63 expression can result from increased Sox2 levels [29], [30], a transcription factor involved in foregut separation that is normally highly expressed in the oesophagus and dorsal part of the trachea but downregulated in the ventral part of the developing trachea, we also monitored Sox2 expression in these sections. WT tracheas (Figure 7A, 7C, bottom panels) and the partially separated *Asciz* ^−/−^ trachea (Figure 7B, bottom panel) exhibited the expected dorsally polarized Sox2 expression pattern; in contrast, Sox2 was still expressed at high levels throughout the ventral part of the bud-like structure in the *Asciz* ^−/−^ embryo (Figure 7D). Thus, while impaired local down-regulation of Sox2 could contribute to the *Asciz^−/−^* phenotype, it is interesting to note that most of the ectopic Sox2-positive cells in the tracheal bud-like structure were still able to downregulate p63. We also observed aberrantly high Sox2 levels in the ventral foregut in *Asciz* ^−/−^ embryos around E10.25, i.e. before oesophagus and trachea were separated in the matched littermate control with appropriately down-regulated Sox2 (Figure S6), indicating that impaired dorso-ventral patterning of Sox2 expression is not merely a secondary consequence of impaired foregut separation in our mutant.

Altogether, these analyses indicate that *Asciz*-deficient mice are able to initially specify the respiratory endoderm, based on Nkx2.1 expression, but then fail to remodel the endoderm in a manner required for initiation of lung budding and efficient separation of the trachea.

### The ASCIZ SQ/TQ-cluster domain has the propensity to activate transcription

**Figure 8:**
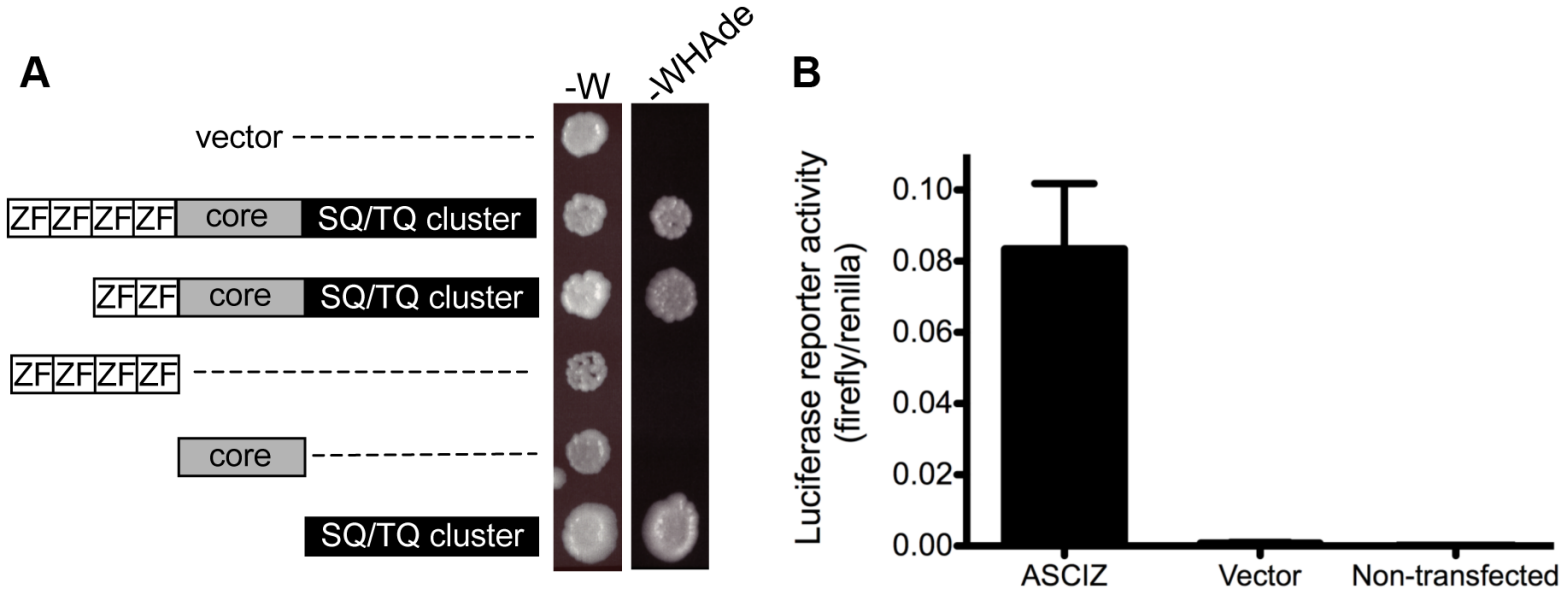
ASCIZ has transcription activating function in reporter assays. (A) Yeast one-hybrid assay. Yeast strains containing the empty vector expressing the Gal4-DBD only or the indicated human ASCIZ constructs (“long form”, residues 1–823; “short form”, residues 156–823; ZnF domain, residues 67–223; “core domain”, residues 230–442; SQ/TQ cluster domain, residues 432–823) were spotted onto -W plates as a loading control and -WHAde plates as an assay for activation of the *GAL1-HIS3* and *GAL2-ADE2* reporter genes. (B) Dual luciferase reporter assay of human U2OS cells transfected with pCDNA3-Gal4DBD or Gal4DBD-ASCIZ667.

When ASCIZ was originally isolated in a yeast two-hybrid screen [15], we noticed during vector-swapping control experiments that ASCIZ could very strongly activate yeast two-hybrid reporter genes on its own once it was fused to the Gal4 DNA-binding domain (Gal4-DBD). As a large proportion of genes that regulate foregut development function as transcription factors (e.g., Sox2, p63, Nkx2.1 mentioned above), and because the modular domain composition of ASCIZ resembles some ZnF transcription factors (see below), we revisited the yeast reporter system to explore the potential of ASCIZ to function as a transcriptional regulator. Both the four-ZnF 823-residue and the two-ZnF 667-residue splice isoforms of human ASCIZ were able to activate the *GAL1-HIS3* and *GAL2-ADE2* reporter genes in these one-hybrid assays (Figure 8A). Importantly, similar dual luciferase reporter assays in human U2OS cells using the 667-residue isoform demonstrated that ASCIZ also has an intrinsic ability to activate gene expression in mammalian cells when tethered to promoters (Figure 8B). Interestingly, truncation analysis revealed that the SQ/TQ-cluster domain - but not the ZnF or core domains - of ASCIZ was sufficient for reporter gene activation (Figure 8A).

## Discussion

### DNA damage and ATM-related functions of ASCIZ

Here we have shown that *Asciz* is essential for pulmonary organogenesis during embryonic development in mice, and required for proper DNA base damage responses in primary cells. Although the lung defect is mechanistically most likely unrelated to defective DNA damage responses, the overall phenotype - MMS and H_2_O_2_ hypersensitivity and embryonic lethality - is consistent with a role of ASCIZ as an accessory BER factor downstream of glycosylases, as proposed by previous work in human and chicken cells [15], [16]. Although *Asciz* null embryos die a few days earlier and their lung defect is considerably more severe than in case of *Polß*-deficient embryos, the latter also seem to have a very comparable late gestational growth retardation [10], [11], and furthermore, the essential requirement for *Polß* is also not suppressed by deletion of *p53* [9]. Likewise, embryos deficient in *Yb-1*, another protein recently linked to accessory functions in the BER pathway [31], [32], also share overall similar late embryonic growth retardation and lethality, frequent exencephaly and modestly increased cellular oxidative stress-induced senescence phenotypes [33].

In contrast to similarities with BER-related genes, the phenotype of *Asciz*-deficient mice differs fundamentally from the phenotype of *Atm*-deficient mice. For example, the key phenotype of *Asciz*-deficient mice - embryonic lethality with absence of lungs - is not shared by *Atm*-null mice [20], and the key phenotype of *Atm*-deficiency - dramatically increased ionizing radiation sensitivity - is not shared by *Asciz*-deficient cells [16], [19]. Consistent with normal ATM protein levels in human, mouse or chicken cells in the absence of ASCIZ, ATM signaling was also unaffected in our *Asciz*-deficient MEFs or *Asciz*-depleted human cell lines (Figures S3, S4, and data not shown), including in response to HU, hypotonic NaCl and chloroquine, that required ASCIZ for ATM activation according to Kanu and Behrens [19]. Thus, the completely different phenotypes and absence of ASCIZ effects on ATM stability and activation question the classification of ASCIZ as an “essential co-factor” and regulator of ATM [19]. It is not clear why the other group obtained different results, as our gene targeting strategy was identical to theirs. Kanu and Behrens did not provide genetic background information for their mice, but given that we consistently observed unimpaired ATM levels in *Asciz*-deficient human, chicken or mouse cells, it seems unlikely that the differing effects could be mouse strain-dependent. As we have confirmed normal ATM levels directly in freshly prepared tissue extracts, we can also exclude the possibility that we may have missed differences in protein levels as a result of variable cell culture conditions. Likewise, given that we did not see a meaningful correlation between ATM and ASCIZ levels in numerous independent AT cell lines, including isogenic AT cell controls reconstituted with WT *Atm*, as well as genuine mouse and chicken *Atm* gene deletions (Figure 4), we can only speculate that the previously reported dramatic loss of ASCIZ may be a peculiarity of that particular AT cell line, possibly due to increased genome instability of AT cells. Considering that the positions of 15 potential ATM phosphorylation sites are exactly conserved from chicken to human and mouse ASCIZ, we favour a model where DNA damage-related functions of ASCIZ may be modulated by its direct phosphorylation by ATM. Indeed, our preliminary data that ASCIZ can be directly phosphorylated by ATM *in vitro* and that its MMS-induced focus formation *in vivo* seems to be at least partially regulated by ATM (to be reported elsewhere in detail) are consistent with a functional interaction between the two proteins.

### Role of ASCIZ in lung development

**Figure 9:**
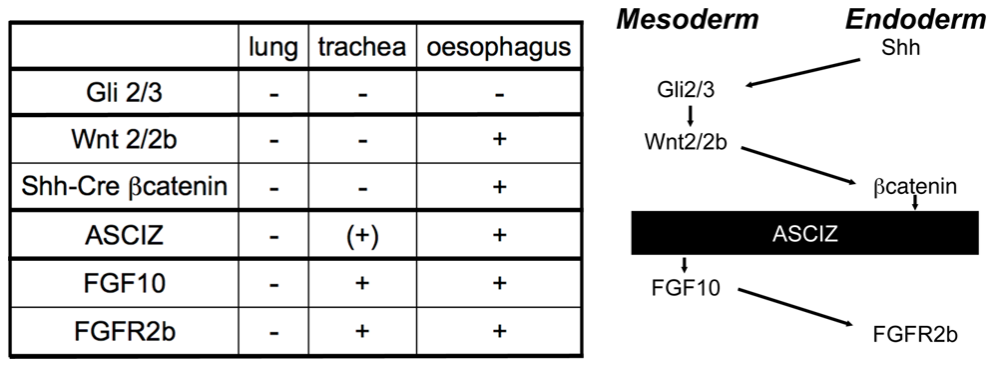
Comparison of the *Asciz^−/−^* phenotype to other mouse mutants with pulmonary agenesis. Table summarizing comparable phenotypes and schematic diagram of their role in the crosstalk between endodermal and mesodermal signaling pathways regulating early respiratory tract development; see discussion for details.

As early lung development is unlikely to be specifically affected by DNA damage signaling, the finding of complete pulmonary agenesis and severe tracheal atresia in *Asciz* null embryos was surprising, particularly as there are very few mouse mutants with comparable respiratory defects (reviewed in [27], [28], [34], [35]). Specification and early development of the respiratory tract is regulated by extensive signaling crosstalk between the foregut endoderm and surrounding mesoderm [27], [28], and mouse mutants have revealed major signaling pathways involved in these processes (Figure 9). Double-knockout mice lacking the Gli2 and Gli3 transcription factors of the hedgehog pathway also seem to lack lungs as well as the trachea; however, they also lack the oesophagus indicating a more severe foregut defect [36](NB, these defects are considerably less severe in sonic hedgehog (*Shh*) null embryos [37]). Foregut development appears overall normal in *Wnt2/Wnt2b* double-null embryos as well as *Shh-Cre* driven conditional *ß-catenin* (*Ctnnb1*) KO mice, but these never establish the Nkx2.1-positive respiratory endoderm and consequently exhibit complete lung and tracheal agenesis [38], [39]. Mice lacking FGF-10 [40], [41] or its cognate FGF-receptor 2b [42] also lack lungs, but seem to contain a grossly normal trachea (and are also characterized by a complete absence of limbs in contrast to the *Asciz^−/−^* phenotype). Conversely, *FoxG1-Cre* driven conditional *Bmp4* deletion results in selective tracheal agenesis, where the main bronchi and primitive lungs emerge directly from the oesophagus [43].

Based on these comparisons (Figure 9), the *Asciz^−/−^* phenotype is less severe than the complete respiratory precursor defect with absence of Nkx2.1 expression and combined agenesis of lungs and trachea in *Wnt2/2b* and *Shh-Cre/ß-catenin* mutants, but more severe than the respiratory tract defect in *Fgf10* or *FGF-receptor 2b* mutants with selective pulmonary agenesis yet preserved tracheal development. Genetically, these data thus suggest a crucial regulatory function for ASCIZ in the regulation of respiratory organogenesis at a level between endodermal ß-catenin and mesodermal FGF10 signaling pathways (Figure 9). As FGF10 has been proposed to regulate the downregulation of Sox2 expression in respiratory precursors [30], our finding of impaired dorso-ventral patterning of Sox2 expression in *Asciz* ^−/−^ embryos before foregut separation (Figure S6), and when tracheal separation stalls early (Figure 7D), are also consistent with a role of ASCIZ upstream of FGF10.

The signaling pathways discussed here ultimately regulate developmentally important gene expression programs during specification, morphogenesis and differentiation of the respiratory system. ASCIZ is a predominantly nuclear protein [15], [19], its ZnF structure is generally reminiscent of transcriptional regulators [44], and we have shown here that ASCIZ has the propensity to function as a transcriptional activator via its SQ/TQ cluster domain (Figure 8). In some regards, ASCIZ can be considered as a mirror image of the Sp1 transcription factor. Whereas ASCIZ contains a ZnF domain at the N-terminus and an extended SQ/TQ cluster towards the C-terminus, Sp1 that also is essential for murine development [45] contains an SQ/TQ rich N-terminal transcription activation domain and a triple-ZnF domain at the C-terminus. While Sp1 has been extensively studied as a transcription factor, it is now becoming apparent that it also has transcription-independent roles as an ATM substrate that relocates into DNA damage-induced foci [46], [47], somewhat similar to our original interest in ASCIZ. Altogether, these analogies make it tempting to speculate that ASCIZ may regulate pulmonary development as a transcription factor.

In conclusion, we have shown here that ASCIZ has dual functions with a role in the response to DNA lesions that are repaired by the BER pathway, as well as pleiotropic functions during murine embryonic development, most notably as a member of a very select group of essential regulators of respiratory organogenesis. Nkx2.1-positive respiratory precursors seem to still be specified in the absence of *Asciz*, but then fail to properly segregate within the foregut. Impaired foregut separation in *Asciz* ^−/−^ embryos seems to correlate with an inability to downregulate Sox2 expression in the ventral foregut, but the exact mechanism responsible for this defect remains to be determined. *Asciz* null embryos die a few days before birth rather than perinatally from an acute inability to breathe, indicating that additional developmental defects beyond the respiratory system may contribute to the lethality. Our study provides a basis to further investigate how exactly ASCIZ regulates respiratory organogenesis and possibly other developmental processes by expanding the analysis to tissue-specific or temporally regulated conditional knockout systems.

## Methods

### Ethics statement

All mouse procedures were approved by the St. Vincent's Hospital Animal Research Ethics Committee.

### Generation and genotyping of *Asciz* gene-targeted mice

The mouse *Asciz* gene contains four exons spread over ∼15 kbp on chromosome 8 and was targeted in C57BL/6 ES cells by integrating loxP sites on both sides of exon D (Figure 1B) using standard homologous recombination, ES cell and blastocyst manipulation techniques as a contracted service by Ozgene Pty Ltd, Perth. A diagnostic *Sca*I restriction site was integrated just 3′-terminal of the downstream loxP site. Gene targeting was confirmed by Southern blotting using 5′- and 3′-probes located outside the targeting vector. The 5′-probe can be used for Southern blot analysis of *Sca*I-digested DNA to distinguish between the WT (22.5 kbp), targeted (11.5 kbp), and *Asciz*-KO (7.6 kbp) alleles (Figure 1B). The germline *Asciz* KO allele was generated by crossing the targeted line with C57BL/6 mice containing a *PGK-Cre* knockin in the *ROSA* locus, followed by two C57BL/6 backcrosses to remove *PGK-Cre* and for embryo transfer into the St. Vincent's Hospital Biological Resources Centre. Thus, the *Asciz* KO line is on a pure C57BL/6 genetic background. Animals were housed in SPF microisolators. Genotyping can also be performed by PCR using primers F1 (5′-CATGGAATTGTTAAAAGCTC-3′), F2 (5′-CCGACTGGGGATGTAGTCAG-3′) and R1 (5′-AAAAGATAGAATAGCTACAC-3′), which result in bands of 170 bp for the WT and 220 bp for the KO allele.

*Asciz^+/−^* mice were crossed with germline *p53*-targeted mice (deletion of exon 2–10 [48], [49]) to generate compound heterozyotes, and offspring were genotyped at weaning using primers above and *p53* primers *Trp53*-1F (5′-CACAAAAAACAGGTTAAACCCAG-3′), *Trp53*-1R (5′-AGCACATAGGAGGCAGAGAC-3′) and *Trp53*-10R (5′-GAAGACAGAAAAGGGAGGG-3′), which result in bands of 290 bp for the WT and 612 bp for the KO allele. Recovery of *p53^−/−^* mice at weaning was approximately half of the expected Mendelian ratios, a known phenomenon for *p53* null homozygosity on inbred backgrounds [50].

### Embryo analyses

The time of pregnancies was defined as E0.5 on the morning vaginal plugs were observed in *Asciz^+/−^* intercrosses. Embryos were dissected from the uterus in cold PBS, weighed after blotting off excess fluid and immediately fixed for histology, or processed for protein extraction or MEF isolation, and genoyped by PCR using yolk sac or tail DNA. For histology, whole embryos were fixed in Bouin's solution or paraformaldehyde, processed to paraffin and sagittal sections were stained using haematoxylin-eosin and scanned using a Zeiss Mirax Digital Slide Scanner by the Australian Phenomics Network Histopathology and Organ Pathology Service, University of Melbourne, or manually processed and photographed as described [51].

### Cell lines and MEF cultures

Human and chicken cell lines were cultured as described [15], [16], [24], [52]. MEFs were prepared by dissecting embryos in cold PBS, heads and internal organs were removed, and remaining corpses were sliced into smaller pieces and trypsinized cells were cultured in Dulbecco's Modified Eagles medium containing 10% fetal calf serum for 2 days, trypsinized, Coulter-counted, and re-seeded at 10^6^ cells per 10 cm dish. This passage, defined as P1, was incubated for 3 days, trypsinized, counted and re-seeded at 10^6^ cells per 10 cm dish (P2), and this process was repeated for 8 passages. For DNA damage sensitivity assays, 5×10^4^ MEFs (P2–P3) were seeded per 35 mm well, grown in Dulbecco's modified Eagle's medium and treated as indicated in the figure legend, and after 18 hours cell viability was determined by propidium iodide exclusion using flow cytometry. Each set of DNA damage sensitivity experiments was performed in parallel with MEFs from at least three independent embryos per genotype.

### Optical projection tomography (OPT)

Staged embryos were stained for OPT [53] with an antibody to E-cadherin (ECCD2, Invitrogen, 1/200 dilution) as described [54], with 48 hour primary and secondary antibody incubations interspersed with extensive 12 hour washes to remove unbound antibody. Samples were imaged on a Bioptonics 3001 OPT machine (Bioptonics, UK) and datasets reconstructed by NRecon (Skyscan, Belgium) and visualized using Drishti (http://anusf.anu.edu.au/Vizlab/drishti/). Embryos were rescued from agarose after imaging, processed to paraffin and sectioned, or directly prepared for cryo-sectioning. After antigen retrieval in citrate buffer sections were stained with antibodies to Nkx2.1 (anti-TTF1, Zymed, 1/200), p63 (Abcam, 1/200), and Sox2 (Chemicon) to examine differentiation.

### Blot analyses

Southern, Northern and Western blots were performed as described [15], [51]. Antibodies against ASCIZ ([15], available from Millipore) and chicken ATM [52] were described before. Other antibodies: Actin (MAB1501, Millipore), ATM (5C2, Abcam), ATR (sc-1887, Santa Cruz Biotechnology), human p53 (sc-126, Santa Cruz Biotechnology), mouse p53 (1C12, Cell Signaling Technology, PML (sc-5621, Santa Cruz), XRCC1 (sc-11429, Santa Cruz), γH2AX (05-636, Millipore), pS1981(mouse: pS1987)-ATM (200-301-400, Rockland; or 10H11.E12, Cell Signaling Technology), pS15(mouse: pS18)-p53 (9284, Cell Signaling Technology), pT68-Chk2 (2661 or 2197, Cell Signaling Technology).

### Transcription reporter assays

For yeast assays, ASCIZ constructs were cloned in pAS2.1 and transformed into PJ69-4A, except the isolated SQ/TQ cluster domain that was cloned into the low-level expression vector pGBT9 because its high level expression was toxic in yeast. One-hybrid reporter assays were performed essentially as described previously for two-hybrid assays in our laboratory [55], [56] except that plates were supplemented with leucine. For mammalian dual luciferase reporter assays, the 667-residue ASCIZ isoform was cloned into pCDNA3-Gal4DBD for transient transfection of U2OS cells with equal amounts of the reporter vectors pFR-Luc and pRL-CMV for use with the Dual-Luciferase Reporter Assay kit (Promega) according to the manufacturer's instructions and measurement of luminescence using a Polarstar Optima (BMG Labtechnologies).

## Supporting Information

Figure S1 Instability of the residual *Asciz* mRNA in *Asciz* null embryos. Northern blot analysis of E14.5 head extracts of 4 separate WT and *Asciz* null embryos probed with exon C- or D-specific probes and *Gapdh* as loading control. Markers on the left indicate (from top to bottom) 10 kb, 8 kb, 6 kb, 4 kb and 3 kb. Note that 2 bands of ∼5.5 kb and ∼3 kb are detected with both *Asciz* probes in the WT, indicating alternative splicing. The similar size of the main band of the exon D-deleted transcript to the 5.5 kb WT mRNA is likely due to read-through from the exon C splice donor junction (in the absence of an exon D splice acceptor) to a poly-adenylation signal downstream of the loxP site. Image quant phosphoimager density units for these bands are: WT, 21545±1282; KO, 2986±1032.
(0.48 MB TIF)

Figure S2 *Asciz*-deficiency only partially impairs base excision repair. Primary MEFs (5–6 embryos per genotype; independent preparations from those shown in Figure 3) were pretreated with 6 mM methoxyamine (MOA) for 2 hours and then with 0.005% MMS for 18 hours as indicated before propidium iodide exclusion assay by FACS.
(4.45 MB TIF)

Figure S3 Unimpaired ATM signaling in *Asciz^−/−^* MEFs. (A) Western blot analysis of WT and *Asciz*-deficient primary MEF cultures treated with 0.01% MMS or 40–120 µg/ml choloroquine (CHQ) for 4 hours, probed with the indicated antibodies. (B) Western blot analysis of WT and *Asciz*-deficient primary MEF cultures treated for 4 hours with 0.01% MMS, 2 mM HU or 20 µg/ml bleomycin, or for 1 hour with 50 mM NaCl, probed with the indicated antibodies (top panels); identical experiments except that MMS treatment was for only 1 hour (bottom panels). (C) Western blot analysis of WT and *Asciz*-deficient primary MEF cultures treated with 0.025% MMS for 3 hours and 15 µM KU55933 (ATMi; with pretreatment for 2 hours before MMS addition) as indicated.
(0.82 MB TIF)

Figure S4 Unimpaired ATM signaling in *Asciz*-depleted human U2OS cells. (A) U2OS cells were treated with *GL2* control or *Asciz* siRNA si579 [15] and treated with 0.02% MMS for the indicated times. (B) U2OS cells were treated with *GL2* control or two separate *Asciz* siRNAs as described and lysed 1 hour after 2 Gy gamma irradiation, and blotted with the indicated antibodies. The arrow points to the position of ATM in the pS1981-ATM blot, the more abundant upper band represents cross-reactivity of the antibody with near-identical phosphorylation sites in a larger protein, possibly 53BP1. Blots above and below the lines are from separate experiments. Note that an older ASCIZ antibody batch was used for this experiment that crossreacts with a ∼100 kDa band just below ASCIZ not observed with the new antibody batch in the other figures.
(0.27 MB TIF)

Figure S5 Additional embryo analyses. E12.5 WT and *Asciz^−/−^* littermates were stained with E-cadherin for whole-mount optical projection tomography similar to Figure 6.
(0.29 MB TIF)

Figure S6 Analysis of marker expression before foregut separation. Cryo-sections of E10.25 WT and *Asciz* ^−/−^ littermates stained with the indicated antibodies. Panels are oriented with the dorsal foregut on top.
(2.00 MB TIF)
