# Correction: Dual Functions of ASCIZ in the DNA Base Damage Response and Pulmonary Organogenesis

**DOI:** 10.1371/annotation/bce92e15-60b4-493b-89de-28529386f4ac

**Published:** 2010-11-17

**Authors:** Sabine Jurado, Ian Smyth, Bryce van Denderen, Nora Tenis, Andrew Hammet, Kimberly Hewitt, Jane-Lee Ng, Carolyn J. McNees, Sergei V. Kozlov, Hayato Oka, Masahiko Kobayashi, Lindus A. Conlan, Timothy J. Cole, Ken-ichi Yamamoto, Yoshihito Taniguchi, Shunichi Takeda, Martin F. Lavin, Jörg Heierhorst

There was an error in affiliation 7 for author Martin F. Lavin. Affiliation 7 should be: Centre for Clinical Research, University of Queensland, Royal Brisbane Hospital, Herston, Australia.

