# Supplementary figures and images for: Dual Functions of ASCIZ in the DNA Base Damage Response and Pulmonary Organogenesis
Source: PLoS Genet. 2010 Oct 21;6(10):e1001170. doi: 10.1371/journal.pgen.1001170 (PMC2958817; doi:10.1371/journal.pgen.1001170)

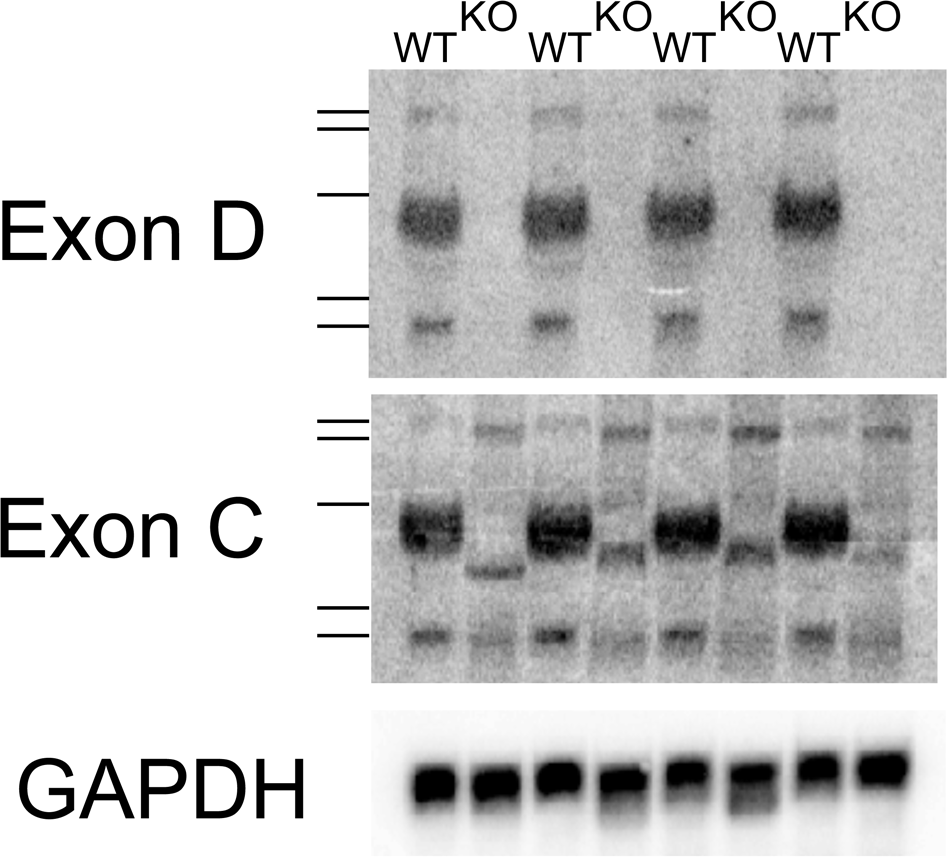

Supplement: Figure S1 — Instability of the residual Asciz mRNA in Asciz null embryos. Northern blot analysis of E14.5 head extracts of 4 separate WT and Asciz null embryos probed with exon C- or D-specific probes and Gapdh as loading control. Markers on the left indicate (from top to bottom) 10 kb, 8 kb, 6 kb, 4 kb and 3 kb. Note that 2 bands of ∼5.5 kb and ∼3 kb are detected with both Asciz probes in the WT, indicating alternative splicing. The similar size of the main band of the exon D-deleted transcript to the 5.5 kb WT mRNA is likely due to read-through from the exon C splice donor junction (in the absence of an exon D splice acceptor) to a poly-adenylation signal downstream of the loxP site. Image quant phosphoimager density units for these bands are: WT, 21545±1282; KO, 2986±1032. (0.48 MB TIF) [file pgen.1001170.s001.tif]

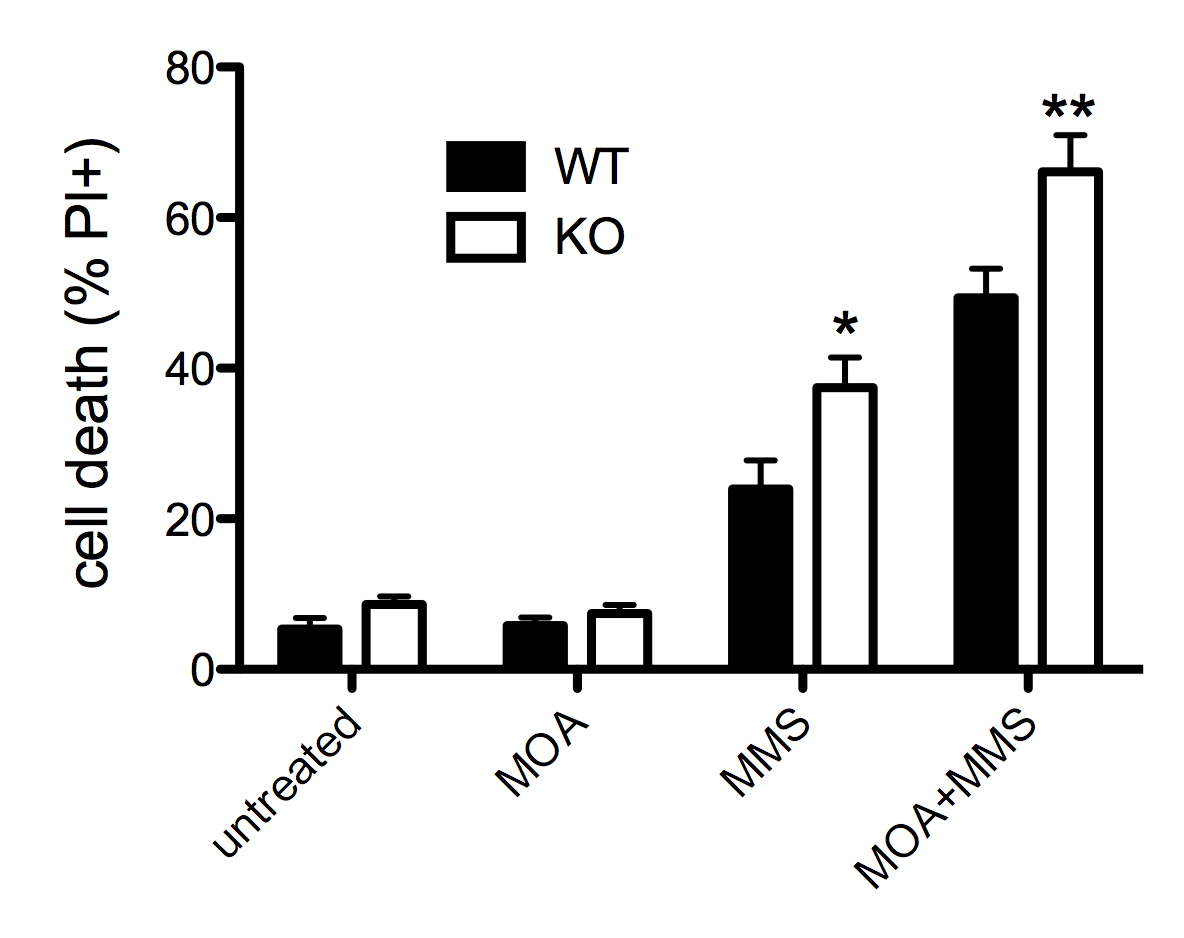

Supplement: Figure S2 — Asciz-deficiency only partially impairs base excision repair. Primary MEFs (5–6 embryos per genotype; independent preparations from those shown in Figure 3) were pretreated with 6 mM methoxyamine (MOA) for 2 hours and then with 0.005% MMS for 18 hours as indicated before propidium iodide exclusion assay by FACS. (4.45 MB TIF) [file pgen.1001170.s002.tif]

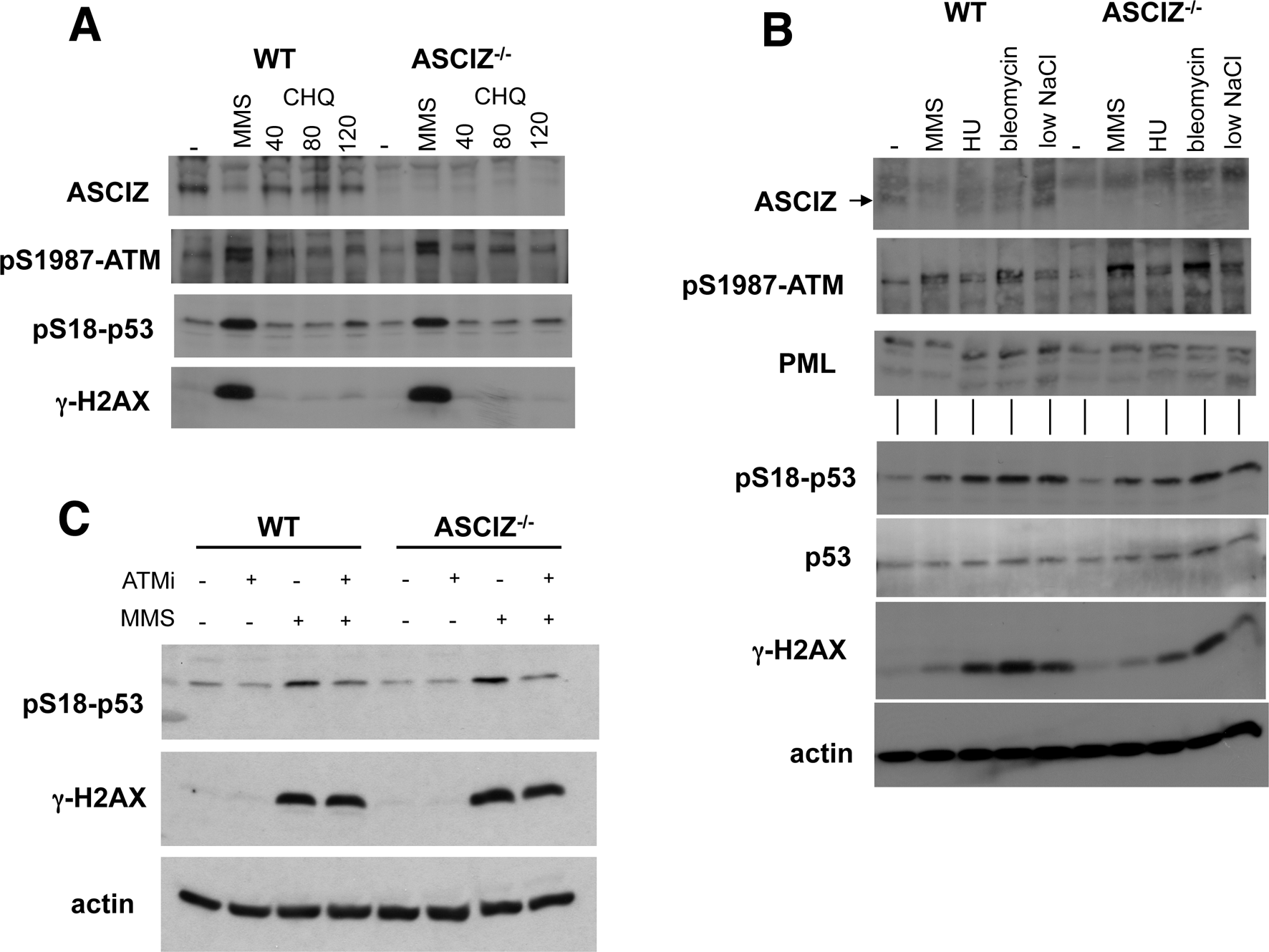

Supplement: Figure S3 — Unimpaired ATM signaling in Asciz−/− MEFs. (A) Western blot analysis of WT and Asciz-deficient primary MEF cultures treated with 0.01% MMS or 40–120 µg/ml choloroquine (CHQ) for 4 hours, probed with the indicated antibodies. (B) Western blot analysis of WT and Asciz-deficient primary MEF cultures treated for 4 hours with 0.01% MMS, 2 mM HU or 20 µg/ml bleomycin, or for 1 hour with 50 mM NaCl, probed with the indicated antibodies (top panels); identical experiments except that MMS treatment was for only 1 hour (bottom panels). (C) Western blot analysis of WT and Asciz-deficient primary MEF cultures treated with 0.025% MMS for 3 hours and 15 µM KU55933 (ATMi; with pretreatment for 2 hours before MMS addition) as indicated. (0.82 MB TIF) [file pgen.1001170.s003.tif]

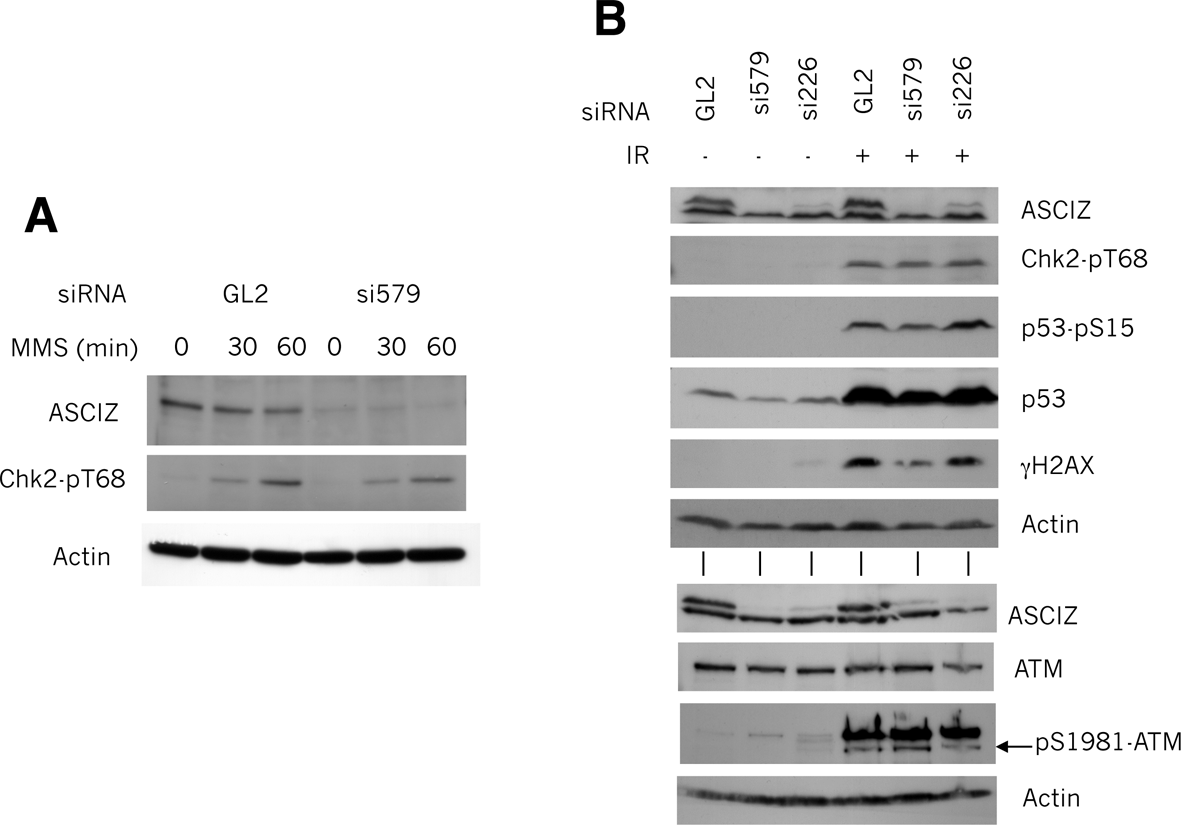

Supplement: Figure S4 — Unimpaired ATM signaling in Asciz-depleted human U2OS cells. (A) U2OS cells were treated with GL2 control or Asciz siRNA si579 [15] and treated with 0.02% MMS for the indicated times. (B) U2OS cells were treated with GL2 control or two separate Asciz siRNAs as described and lysed 1 hour after 2 Gy gamma irradiation, and blotted with the indicated antibodies. The arrow points to the position of ATM in the pS1981-ATM blot, the more abundant upper band represents cross-reactivity of the antibody with near-identical phosphorylation sites in a larger protein, possibly 53BP1. Blots above and below the lines are from separate experiments. Note that an older ASCIZ antibody batch was used for this experiment that crossreacts with a ∼100 kDa band just below ASCIZ not observed with the new antibody batch in the other figures. (0.27 MB TIF) [file pgen.1001170.s004.tif]

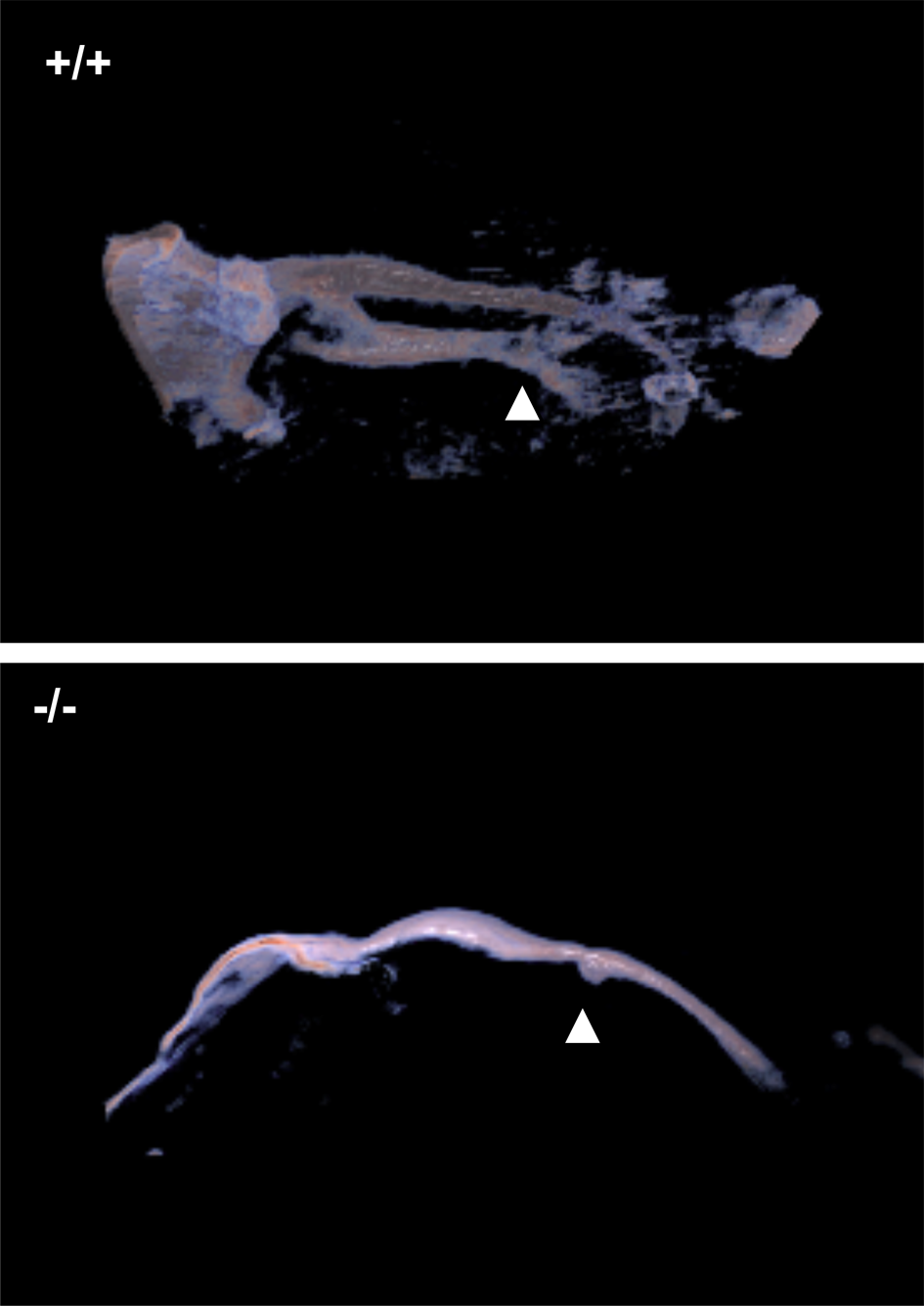

Supplement: Figure S5 — Additional embryo analyses. E12.5 WT and Asciz−/− littermates were stained with E-cadherin for whole-mount optical projection tomography similar to Figure 6. (0.29 MB TIF) [file pgen.1001170.s005.tif]

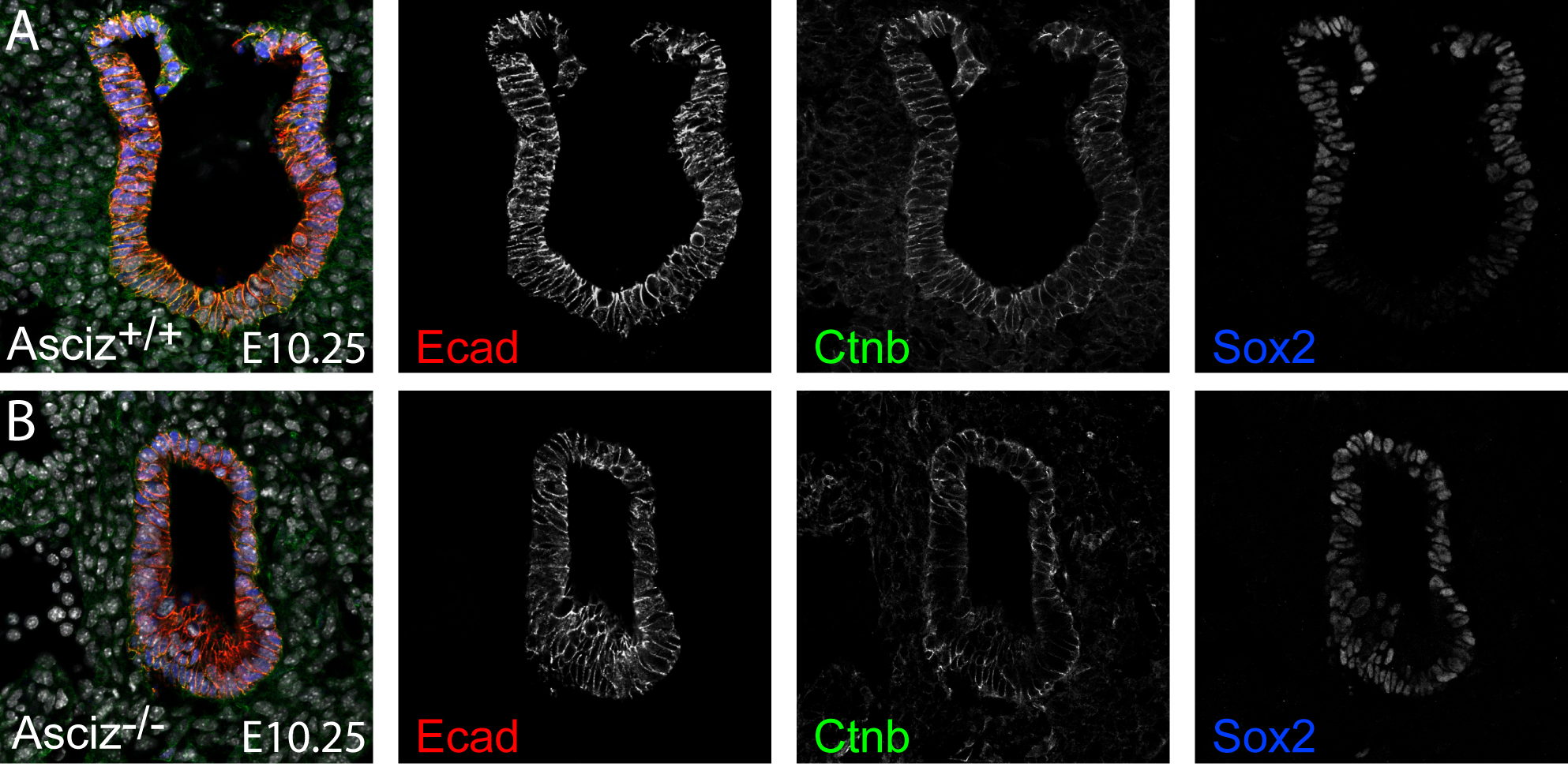

Supplement: Figure S6 — Analysis of marker expression before foregut separation. Cryo-sections of E10.25 WT and Asciz −/− littermates stained with the indicated antibodies. Panels are oriented with the dorsal foregut on top. (2.00 MB TIF) [file pgen.1001170.s006.tif]
